# Stigma Associated With Academic Failure in Nursing Education: A Triangulated Qualitative Study of Nursing Students and Nurse Educators

**DOI:** 10.1002/nop2.70635

**Published:** 2026-06-25

**Authors:** Esther Albertinah Mpangane, Nkhensani Florence Mabunda, Deliwe Rene Phetlhu

**Affiliations:** ^1^ Department of Nursing, Faculty of Health Care Science Sefako Makgatho Health Sciences University Pretoria South Africa

**Keywords:** academic failure, nurse educators, nursing students, qualitative research, stigma, triangulation

## Abstract

**Aim:**

To integrate the perspectives of repeating nursing students, nurse educators, and non‐repeating nursing students to understand how stigma associated with academic failure manifests within nursing education institutions.

**Design:**

Qualitative triangulation study.

**Methods:**

Data were collected across four campuses of a South African nursing education institution using individual semi‐structured interviews with repeating nursing students (*n* = 8) and focus group discussions with nurse educators (*n* = 47; nine groups) and non‐repeating nursing students (*n* = 51; nine groups). Participants were purposively sampled based on their direct experience of the phenomenon. Data were analysed using thematic analysis within each participant group, followed by triangulation to identify convergence, divergence, and complementarity across datasets.

**Results:**

Stigma associated with academic failure operated across interpersonal, structural, and internalised levels. Five integrated themes were identified: (1) stigmatising behaviours and labelling; (2) institutional policies and administrative practices; (3) peer exclusion and professional conduct; (4) psychological and financial consequences; and (5) coping responses and resilience. Participants described public identification as ‘repeaters’, inequitable treatment, financial hardship after stipend withdrawal, diminished confidence, anxiety, and social isolation.

**Conclusion:**

Stigma associated with academic failure is systemically embedded within nursing education and reinforced through educator practices, peer interactions, and institutional systems. Multilevel interventions are required to foster equitable, psychologically safe, and inclusive learning environments.

**Impact:**

Findings provide evidence to guide educator development, anti‐stigma interventions, student support initiatives, and policy reform within nursing education institutions.

**Patient/Public Contribution:**

No patient or public involvement occurred in the design, conduct, reporting, or dissemination of this study beyond participation of nursing students and nurse educators as research participants.

**Abbreviations**
NEI: nursing education institutions
NEs: nurse educators
NRNS: non‐repeating nursing students
RNS: repeating nursing students
SAWAF: stigma associated with academic failure

## 1 Introduction

Academic failure remains a persistent challenge within nursing education globally and is associated with delayed progression, attrition, financial burden, and psychological distress among students (Lewis 2020; Mpangane et al. 2026; Pierre 2023; Wahid et al. 2021). Students who repeat courses after academic failure may encounter stigma that extends beyond their academic performance. Such stigma may adversely affect confidence, belonging, motivation, and engagement with learning (Douville 2024; Tonelli 2022).

Stigma has been conceptualised as a social process through which individuals are labelled, stereotyped, separated, and disadvantaged within unequal power relations (Andersen et al. 2022; Nyblade et al. 2019). Within educational settings, stigma may manifest as enacted stigma (overt discriminatory behaviours), structural stigma (policies or institutional practices that reinforce disadvantage), and internalised stigma (self‐directed shame or diminished self‐worth). Applying these distinctions enables a clearer understanding of how academic failure becomes socially meaningful and harmful within nursing education.

Emerging evidence suggests that students who fail or repeat nursing courses may experience embarrassment, exclusion, fear of judgement, and reduced academic confidence (Jakubec et al. 2020; Lewis 2020). However, most studies focus on individual student experiences and less frequently include the perspectives of educators and peers who may also shape stigma processes.

Triangulation of multiple stakeholder perspectives can enhance credibility and generate a richer understanding of complex phenomena. Integrating the views of repeating nursing students (RNS), nurse educators (NEs), and non‐repeating nursing students (NRNS) may illuminate how stigma is produced, maintained, and resisted across educational contexts.

**FIGURE 1:**
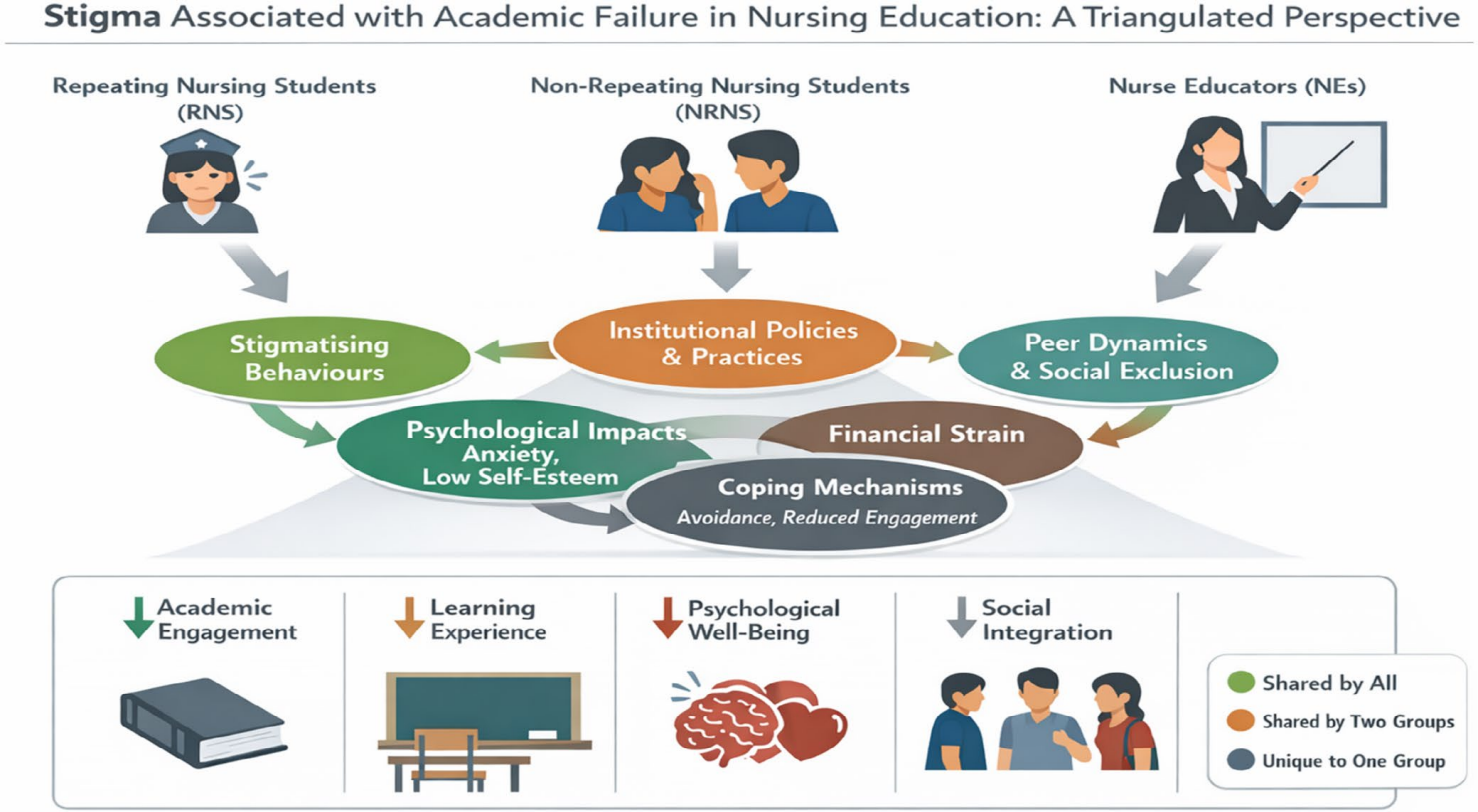
Graphic abstract illustrating the triangulated qualitative design and integrated findings of stigma associated with academic failure (SAWAF) in nursing education. The figure demonstrates how enacted, structural, and internalised forms of stigma emerge across the perspectives of repeating nursing students (RNS), non‐repeating nursing students (NRNS), and nurse educators (NEs). These interconnected processes contribute to psychological distress, financial strain, maladaptive coping responses, and reduced academic engagement.

This study, therefore, aimed to integrate the perspectives of RNS, NEs, and NRNS to understand how stigma associated with academic failure manifests within nursing education institutions. A graphical summary of the triangulated study design and the multilevel manifestations of stigma associated with academic failure is presented in Figure 1.

## 2 Aim

To integrate the perspectives of RNS, NEs, and NRNS to understand how stigma associated with academic failure manifests within nursing education institutions.

## 3 Significance of the Study

This study advances understanding of stigma associated with academic failure as a multidimensional phenomenon within nursing education. By integrating the perspectives of repeating students, peers, and educators, the triangulated design enabled identification of interpersonal, structural, and internalised stigma processes that may not be visible through single‐source studies. Findings provide evidence to guide anti‐stigma interventions, educator development, student support, and equitable policy reform.

## 4 Methods

### 4.1 Design

A qualitative triangulated design was employed to integrate findings from three participant groups and examine converging, diverging, and complementary perspectives on stigma associated with academic failure (SAWAF). The study formed part of a broader doctoral project. This manuscript reports the Phase One qualitative triangulated analysis; subsequent phases focused on intervention development informed by Lewin's Change Theory, including unfreezing, change implementation, and refreezing strategies to reduce SAWAF within nursing education institutions.

### 4.2 Setting

The study was conducted across four campuses of a South African nursing education institution providing undergraduate nursing education and clinical placements.

### 4.3 Participants and Sampling

Purposive sampling was used to recruit participants with direct knowledge or experience of SAWAF. Three participant groups were included:

- RNS who had repeated academic levels or courses following failure (*n* = 8)
- NEs involved in teaching and assessment (*n* = 47)
- NRNS attending classes with RNS (*n* = 51)

Recruitment continued until data saturation was reached within each participant group, defined as the stage at which no substantially new codes, meanings, and insights emerged.

### 4.4 Participant Characteristics

Among RNS, five were male and three female, aged 19–45 years. Two older female participants had entered via Recognition of Prior Learning.

Among NEs, participants ranged from 30–65 years; 37 were female and 10 male. Teaching experience ranged from 2 to 21 years.

Among NRNS, 44 were female and seven male, aged 20–42 years. All were enrolled at Level 3.

### 4.5 Data Collection

Data were collected by the principal investigator. Individual semi‐structured interviews were conducted with RNS because academic failure and stigma were considered sensitive personal experiences better explored privately. Focus group discussions were conducted with NEs and NRNS to examine shared perceptions, social norms, and group dynamics. Data collection comprised:

- Eight individual interviews with RNS.
- Nine focus groups with NEs.
- Nine focus groups with NRNS.

Interview sessions lasted approximately 45–60 min, were audio‐recorded with consent, and transcribed verbatim. No prior supervisory or evaluative relationship existed between the interviewer and participants.

### 4.6 Data Analysis

Thematic analysis was undertaken in six stages: familiarisation, coding, searching for themes, reviewing themes, defining themes, and reporting findings. Each participant dataset was first analysed independently. Thereafter, triangulation compared themes across the three datasets to identify:

- Convergence (shared findings)
- Divergence (contrasting perspectives)
- Complementarity (findings that extended understanding)

This process enabled the construction of integrated higher‐order themes. To illustrate how findings from the three participant groups were integrated during triangulation, a summary of dataset‐specific findings is presented in Table 1.

**TABLE 1:** Summary of dataset‐specific findings integrated during triangulation across the three participant groups.

Qualitative research data		
Objective one	Objective two	Objective three
To explore the experiences of RNS regarding the stigma associated with academic failure at a NEI in S.A.	To explore the perceptions of NEs regarding the stigma associated with academic failure among RNS at a NEI in S.A.	To explore the perceptions of NRNS regarding attending classes with RNS at a NEI in S.A.
**Key findings statements**	**Key findings statements**	**Key findings statements**
**1**. Unnecessary pressure from NEs towards the RNS to answer questions regardless of preparedness.	**13**. Unwarranted higher academic expectations for RNS than NRNS by NEs.	**27**. Unrealistic academic expectations are placed on RNS by NEs.
**2**. Reduced academic status of RNS to the label of ‘repeaters’ by NEs, especially in front of their NRNS peers, overshadows their identity and personal names.	**14**. Lack of consensus among NEs on whether RNS are being singled out deliberately or unintentionally.	**28**. Stigmatising behaviours by NRN and NEs towards RNS include labelling them as ‘repeaters’, publicly singling them out, and excluding them, which often involve figurative references to the Gautrain (a rapid transit system) that subtly highlight the academic failure of RNS
**3**. Social stigmatisation of RNS by NRNS through gossip and negative comments.	**15**. Acknowledgement of the stigmatising behaviours and confidential breaches by NEs towards RNS.	**29**. Unethical conduct by NEs includes discussing the academic failures of RNS with others, particularly with their NRNS colleagues.
**4**. Stigmatisation resulting from the NEI practice of categorising and or excluding RNS from the class attendance registers.	**16**. Stigmatisation resulting from the NEI practice of categorising RNS as ‘repeaters’ as well as excluding or delaying adding to the class attendance registers further exacerbates their social exclusion.	**30**. Stigmatisation of RNS reinforced by institutional policies and practices, such as publicly disclosing student academic results and classifying RNS within attendance registers under a bolded ‘repeaters’ heading.
**5**. Withdrawal of a stipend forced RNS to leave campus accommodation, self‐fund their academic needs, and face family disappointment along with SAWAF.	**17**. Withdrawal of stipends exacerbates the SAWAF experienced by RNS, leading to increased absenteeism among them.	**31**. The withdrawal of stipends for RNS led to practical challenges with transportation and accommodation, decreased motivation and morale, resulting in poorer academic performance and increased SAWAF, while negative family perceptions of academic failure further reinforced SAWAF following the loss of financial assistance.
	**18**. Self‐isolation and withdrawal among the RNS as a response to SAWAF.	**32**. Self‐isolation and forming of exclusive groups with other RNS, not because NRNS exclude them, but due to their own low self‐esteem and fear of being judged by NRNS.
**6**. Inconsistent and rigid enforcement of NEI policy, a lengthy appeal process, and a lack of effective communication and transparency about policy changes cause anxiety, confusion, and distress among the RNS	**19**. Inconsistent and unrealistic implementation of the NEI policy and procedures, particularly concerning the lengthy process of appeals, publication of academic results, and issuance of termination letters, creates uncertainty and distress among RNS.	
**7**. Undermined self‐worth and confidence of the RNS by the NRNS.		**33**. Lack of trust in RNS by NRNS during group work due to perceived unreliability based on their history of academic failure by NRNS peers.
	**20**. Social exclusion and isolation of RNS in group activities and collaborative activities by their NRNS peers undermine the reputation and learning experience of the RNS.	**34**. Acknowledgement of stigmatisation perpetuated through social isolation and conditional inclusion of RNS in group discussions by the NRNS.
	**21**. Stigmatisation of RNS by clinical staff.	**35**. Stigmatising of RNS extended to the clinical setting
**8**. Self‐blame among RNS for their academic failure.	**22. Self‐blame among** NEs for students' academic failure, seeing it as a reflection on their teaching abilities.	
**9**. Blaming NEs and NEI for the lack of support towards RNS	**23**. Blamed by RNS for NEs' lack of support or perceived dislike due to academic failure, and by NEI for student failures and wasted resources.	
**10**. Psychological impact of SAWAF among RNS encompasses trauma, depression, anxiety, and burnout.	**24**. Psychological impact of SAWAF among RNS, characterised by increased anxiety, panic attacks, and hyperventilation before assessments, due to fear of repeated failure.	
**11**. Persistent self‐doubt and low self‐esteem among RNS.		**36**. Persistent lack of confidence in RNS information among the NRNS peers due to their history of academic failure.
	**25**. Resilience and determination exhibited by some RNS to overcome subsequent academic failure.	**37**. Positive perceptions among NRNS about their RNS peers stem from the collaborative sharing of resources by RNS, which supports the academic success of NRNS.
**12**. Avoidant behaviours exhibited by the RNS as coping strategies in response to SAWAF, which often include avoiding negative comments and withdrawal from academic activities	**26**. Increased alcohol consumption and unprofessional conduct among RNS as maladaptive responses to SAWAF.	**38**. Indirect stigmatising remarks by NEs, such as figurative expressions related to Gautrain (a rapid transit system) that subtly highlight the academic failure of RNS.

### 4.7 Trustworthiness

Credibility was enhanced through triangulation, iterative engagement with the data, and use of verbatim quotations.

Dependability was supported through an audit trail documenting coding and theme development.

Confirmability was strengthened through supervisory review of analytic decisions.

Transferability was supported through a detailed contextual description.

### 4.8 Ethical Considerations

Ethical approval was obtained from the relevant institutional ethics committee and national regulatory authority. Written informed consent was obtained from all participants.

## 5 Results

Five integrated themes were developed through triangulation of convergent, divergent, and complementary findings across the three participant groups (Table 1).

### 5.1 Stigmatising Behaviours and Labelling

Across groups, participants described public identification of RNS as ‘repeaters’, heightened scrutiny, and assumptions of incompetence. One RNS explained:

They no longer called us by our names. We were just the repeaters. (RNS AA)

Another participant stated:

*Even when you are not ready, they expect you to answer because you failed before*. *(RNS AC)*

NEs acknowledged higher expectations towards RNS, often framed as accountability. However, NRNS recognised these practices as unfair and embarrassing for peers.

This theme reflects enacted stigma through labelling, differential treatment, and public marking of failure.

### 5.2 Institutional Policies and Administrative Practices

Participants described structural processes that intensified stigma, including stipend withdrawal, delayed registers, public release of results, and inconsistent appeals procedures. An RNS noted:

*When the stipend stopped, everything became difficult—transport, food, even where to stay*. *(RNS AD)*

NEs also recognised distress caused by prolonged appeals and unclear communication. These findings indicate structural stigma where institutional systems unintentionally deepen disadvantage.

### 5.3 Peer Exclusion and Professional Conduct

RNS described gossip, distancing, and exclusion from group activities. Some NRNS admitted avoiding collaboration with RNS due to concerns about academic reliability. One NRNS stated:

*Some students think if you work with repeaters your marks will go down*. *(NRNS 7)*

Participants also reported breaches of confidentiality by some educators discussing students' results publicly. This theme illustrates how peer culture and professional misconduct sustain stigma.

### 5.4 Psychological and Financial Consequences

RNS reported anxiety, shame, low self‐esteem, fear of repeated failure, and emotional exhaustion. One participant shared:

*Before tests I could not breathe properly. I was scared of failing again*. *(RNS AF)*

Financial strain compounded distress and reduced attendance and concentration. NEs observed panic symptoms and withdrawal among some students.

This theme demonstrates the intersection between internalised stigma and material hardship.

### 5.5 Coping Responses and Resilience

Some RNS coped through avoidance, silence, sitting at the back of classrooms, or limiting participation.

*I stayed quiet, so nobody notices me*. *(RNS AH)*

However, others described determination to succeed despite stigma. NEs also identified resilience among certain students who became more focused after failure. This theme highlights both maladaptive coping and recovery potential.

## 6 Discussion

This study demonstrates that stigma associated with academic failure is not merely an individual emotional response but a socially and institutionally produced phenomenon embedded within nursing education. As illustrated in Figure 1, these findings demonstrate how stigma associated with academic failure operates simultaneously across enacted, structural, and internalised levels within nursing education.

Enacted stigma was evident through labelling, public scrutiny, exclusion, and assumptions of incompetence. Although some educators perceived heightened expectations as motivational, students often experienced these interactions as humiliating or inequitable. Similar findings have been reported in studies of educational discrimination and academic shame (Hadian Jazi et al. 2022; Douville 2024).

Structural stigma emerged through institutional practices such as public categorisation of students, inconsistent administrative processes, and withdrawal of financial support. These systems transformed temporary academic difficulty into a visible social identity. Financial barriers are well documented as contributors to poor academic outcomes in South African higher education contexts (Wildschut et al. 2020).

Internalised stigma was reflected in shame, self‐doubt, withdrawal, and reduced participation. Importantly, stigma therefore appears cyclical: institutional and interpersonal signals are internalised, which then reduce engagement and may contribute to further academic risk.

Peer dynamics played a central role. Distancing from RNS limited collaborative learning opportunities and reinforced outsider status. Yet some peers recognised the academic value and generosity of RNS, suggesting that stigma is neither universal nor inevitable.

The findings also extend to clinical learning settings, where students may receive fewer developmental opportunities because of perceived academic weakness. Such inequity may affect competence development and professional identity formation (Cooper et al. 2025). These findings indicate that multilevel responses involving educators, peers, and institutional systems are required. At the institutional level, policies and administrative practices that publicly identify unsuccessful students should be reconsidered. Financial and counselling supports are also essential. At the peer level, inclusive group learning strategies may reduce exclusion.

Using Lewin's Change Theory, institutions may first unfreeze stigmatising norms through awareness training, then change practices via educator development and policy revision, and finally refreeze inclusive behaviours through monitoring, leadership support, and curriculum integration.

## 7 Limitations

This study was conducted within one South African nursing education institution, which may limit transferability to other contexts. Although triangulation strengthened credibility, participants' responses may have been influenced by recall bias or social desirability. Focus group discussions may also have constrained dissenting opinions. As a qualitative study, findings are contextually situated rather than statistically generalisable. Future multi‐site studies are recommended.

## 8 Conclusion

Stigma associated with academic failure is systemically embedded within nursing education institutions and reinforced through educator practices, peer relations, and institutional systems. Addressing this phenomenon requires coordinated educational, organisational, and relational change to promote equity, dignity, psychological safety, and academic success for all nursing students.

## Author Contributions

**Esther Albertinah Mpangane:** conceptualisation, methodology, investigation, data curation, formal analysis, writing – original draft, writing – review and editing, project administration. **Nkhensani Florence Mabunda:** supervision, validation, writing – review and editing. **Deliwe Rene Phetlhu:** supervision, validation, writing – review and editing.

## Funding

The authors have nothing to report.

## Disclosure

The views expressed in this article are those of the authors and do not necessarily represent the official positions or policies of any affiliated organisations.

No patient or public involvement occurred in the design, conduct, reporting, or dissemination of this study. Nursing students and nurse educators participated solely as research participants.

This study was reported in accordance with the Consolidated Criteria for Reporting Qualitative Research (COREQ) guidelines.

## Ethics Statement

Ethical approval for the study was obtained from the Sefako Makgatho Health Sciences University Research Ethics Committee (SMUREC/H/486/2023:PG) and the National Health Research Database (GP202402115). The study was conducted in accordance with the ethical principles outlined in the Declaration of Helsinki for research involving human participants.

## Consent

All participants were requested to provide informed voluntary consent, obtained both verbally and in writing. Unique codes were generated for data collection purposes to ensure participant anonymity.

## Conflicts of Interest

The authors declare no conflicts of interest.

## Data Availability

The data that support the findings of this study are available from the corresponding author upon reasonable request.
